# Henoch-Schönlein purpura with joint involvement: Analysis of 71 cases

**DOI:** 10.1186/s12969-016-0080-x

**Published:** 2016-03-31

**Authors:** Xuehong Wang, Yongmei Zhu, Laiqiang Gao, Shuyue Wei, Youyou Zhen, Qiang Ma

**Affiliations:** Dongying Hospital of Shandong Provincial Hospital, Dongying, Shandong province 257091 China

**Keywords:** Henoch-Schönlein purpura, Joint involvement, HSP nephritis, Risk factors

## Abstract

**Background:**

Although joint involvement is the second most common clinical manifestation after skin involvement in patients with Henoch-Schönlein purpura (HSP), it has not been well characterized. The aim of this study was to profile the clinical characteristics and identify the potential risk factors for kidney damage in HSP patients having joint involvement.

**Methods:**

We retrospectively reviewed 71 cases of HSP patients with joint involvement who attended our hospital between January 2010 and March 2012 and analyzed their epidemiological profile, clinical characteristics, follow-up findings (up to three years) and overall prognosis. Logistic regression analysis was performed to identify risk factors associated with renal symptoms in HSP patients with joint involvement.

**Results:**

Average age of patients was 8.55 ± 2.13 years with male to female ratio at 1.29:1. The peak age of disease onset was six to 11 years. The most common triggers included upper respiratory infection, vigorous physical activity, and autumn and winter seasons. Forty cases (56.35 %) had gastrointestinal involvement and 37 (52.11 %) had kidney damage; gastrointestinal system, scrotal involvement, and increased D-dimer levels were significantly associated with kidney injury (*P* < 0.05) by multivariate analysis. Glucocorticoid therapy was effective in alleviating symptoms.

**Conclusion:**

Gastrointestinal symptoms, scrotal involvement, and increased D-dimer are the potential risk factors for kidney damage in HSP patients having joint involvement. Rational use of corticosteroids was probably responsible for the good clinical outcomes.

## Background

Henoch-Schönlein purpura (HSP) is the most common form of vasculitis in children. The condition has been known for nearly 200 years. The estimated annual incidence of HSP in China is 14.06 cases per 100,000 children [1] with an increasing trend observed over successive years, however, this rising trend has recently tapered, possibly due to changes in social, economic, health, and environmental conditions [2]. Factors triggering HSP include infections (mainly upper respiratory infection), drugs, food, insect bites and vaccination. Physical conditions, such as exposure to cold are also known to be environmental triggers for the disease onset. The pathogenesis of HSP is related to aberrant deposition of IgA-containing immune complexes [3], which typically affects skin, gut, joints and glomeruli. Among these, complications resulting from kidney lesions are the most severe. Isolated microscopic haematuria and minimal proteinuria are common occurrences in children, with nephrotic syndrome and renal dysfunction also being observed occasionally. The prognosis is, to a large extent, dependent on the severity of renal involvement [4–6].

However, the incidence of joint involvement in HSP patients could be as high as 78 % [7], which is the second most common feature after dermal manifestations [6, 8]. Joint manifestations in HSP tend to be neglected due to their mild nature. Moreover, atypical cases are liable to be misdiagnosed and mistreated. With the incidence increasing, investigation of every potential case and assessment of renal involvement in these patients is important for the successful management of HSP. With the aim of expanding our knowledge about HSP with joint involvement, we retrospectively reviewed the clinical characteristics, prognosis and kidney damage in these HSP patients with joint involvement.

## Methods

### Patient characteristics

A total of 71 patients with HSP who attended the Department of Dermatology at Dongying Hospital, Shandong Province, between January 2010 and March 2012 were included in this study. The patients were evaluated retrospectively. The diagnosis of HSP was based on European League against Rheumatism (EULAR) endorsed consensus criteria for HSP classification [7]. All cases developed skin purpura, acute arthritis or acute arthralgia, accompanied with: (1) abdominal pain: diffuse colicky pain. Some patients had gastrointestinal bleeding and emesis. (2) histopathology: proliferative glomerulonephritis with predominant IgA deposit or typically leucocytoclastic vasculitis with predominant IgA deposit. (3) renal involvement: proteinuria > 30 mmol/mg of urine albumin/creatinine ratio on a spot morning sample or >0.3 g/24 h; presence of red blood cell casts or haematuria; red blood cells casts in the urinary sediment or > 5 red blood cells/high power field or ≥ 2+ on dipstick. Arthritis was defined as arthralgia with limited movement or joint swelling. Arthralgia was defined as joint pain without joint limited movement or swelling.

The inclusion criteria were as follows: (1) age < 14 years old; (2) no history of allergic or hematological disorders; (3) not diagnosed with systemic lupus erythematosus, renal or any other immunological disorders; (4) no active infections such as tuberculosis, hepatitis B and other contagious diseases. The exclusion criteria were: (1) patients with incomplete information; (2) patients unable to comply with the treatment; (3) patients with severe heart, liver, lung, kidney or other organ system diseases.

A total of 131 HSP patients were admitted to our hospital between January 2010 and March 2012. Of these, 79 cases of HSP had joint involvement and were thus considered for inclusion in this study. Among them, two patients were found to be older than 14 years old (41 and 16 respectively), one case had hypothyroidism, and four patients had incomplete data (of these, one was admitted to another hospital, and three patients were lost to follow up). Thus, a total of 71 cases were finally included in the study. The study was approved by the Ethics Committee at the Dongying Hospital.

### Laboratory investigations

All patients underwent laboratory tests such as antinuclear, anti-DNA, anti-ENA antibodies, routine blood and urine tests, fecal occult blood, coagulation profile, anti-streptolysin O titers, C-reactive protein, levels of C3, IgA, IgG, IgM, D-dimer, BMI value, blood pressure, liver function (ALT, AST), kidney function (SCr, BUN) and joint x-ray.

### Statistical analysis

Continuous variables were expressed as mean ± standard deviation (SD) or median (interquartile range). Categorical variables were expressed as percentages. Inter-group differences on univariate analysis were evaluated by *χ*^2^ test. Multivariate logistic regression analysis was performed to determine odds ratio at 95 % confidence interval (CI). All statistical analyses were performed using SPSS 16.0. *P* < 0.05 was considered statistically significant.

## Results

### Characteristics of patients

**Fig. 1:**
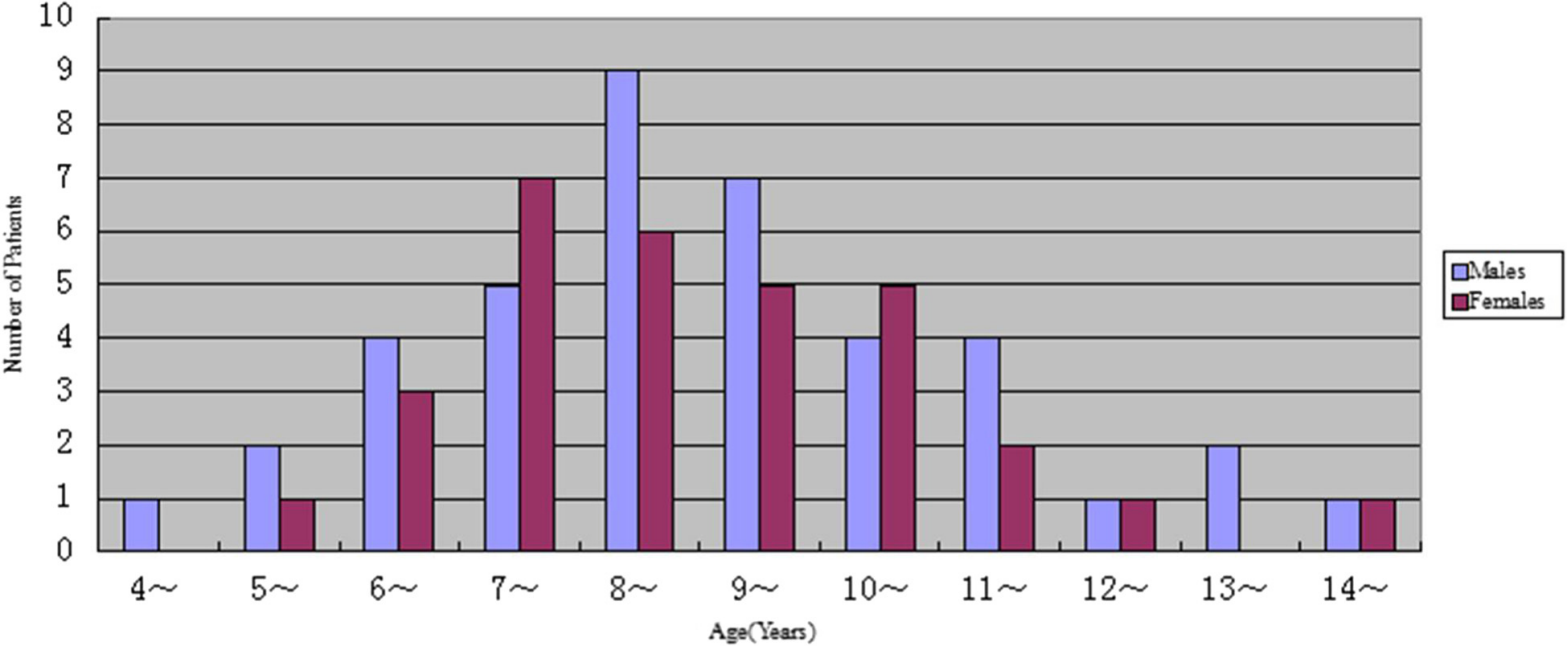
Age and gender distribution of patients. The majority of patients were 6 to 10 years old, mostly male

**Fig. 2:**
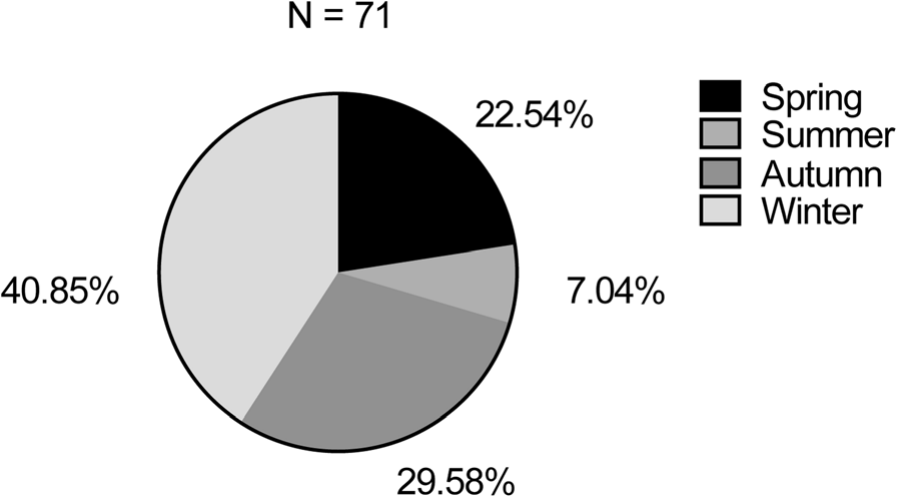
Seasonal distribution of disease onset showing maximum cases with onset in autumn and winter

**Fig. 3:**
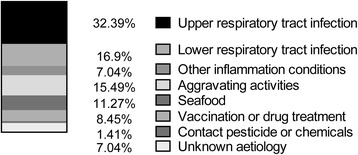
Predisposing factors for HSP. In 49 % patients, the upper and lower respiratory tract infection were the triggers

A total of 71 cases including 40 males and 31 females were included. The ratio of male to female was 1.29:1. The average age was 8.55 ± 2.13 years and median age was eight. Sixty- one cases (86 %) had peak age for disease onset between six and 11 years, with male to female ratio of 1.22:1 (Fig. 1). As shown in Fig. 2, disease onset occurred during all the seasons, with the highest incidence being reported during autumn and winter seasons (70 %). Respiratory tract infection was the most frequent predisposing factor followed by vigorous exercise, with 66 cases (93 %) being aware of the trigger factors (Fig. 3).

### Clinical manifestations

**Fig. 4:**
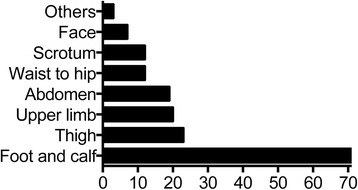
Distribution of skin rash. Foot and lower leg was the most commonly affected area

Main clinical features of the 71 HSP children were summarized in Table 1. All 71 cases had typical skin manifestations such as petechiae and ecchymosis. In 64 cases (90 %), skin symptoms were the initial manifestations. Skin rash was most commonly observed in the lower limbs and foot, followed by thigh, upper limbs, abdomen, waist-hip, scrotum and face. Twenty-seven cases (38 %) had rash in more than three affected areas (Fig. 4).

**Table 1:** Clinical features of HSP patients with joint involvements (*N* = 71)

Symptom	N	Percent
Skin rash	71	100
Joint manifestations	71	100
Arthritis	62	87
Arthralgias	9	13
Gastrointestinal manifestations	40	56
Abdominal pain	35	49
Vomiting	21	30
Gastrointestinal bleeding	13	18
Renal involvement	37	52
Acute nephritis	31	44
Hematuria and proteinuria	13	18
Nephrotic syndrome	11	15
Proteinuria	8	11
Hematuria	6	8
Chronic nephritis	2	3
Leg edema	12	17
Fever	7	10
Scrotum pain	5	7
Nervous system involvement	3	4
Lung involvement	2	3

### Purpura

Petechiae and ecchymosis were distributed symmetrically interspersed with mild raised skin bumps spots. The colour was bright crimson to begin with, and then turned dark and faded over a period of three to ten days. Three children developed wheal-like rash, necrotizing purpura, blisters or bullae. 52 (73 %) patients experienced recurrent skin symptoms. Twenty-nine cases (41 %) relapsed within 1 month, 15 cases recurred within 1 to 3 months (21 %), six cases (8 %) recurred in 3 months to half a year, and two cases (3 %) relapsed after 8 months and 14 months respectively. 22 cases (31 %) had a recurrence of skin lesions on more than three occasions.

### Joint symptoms

Most children developed joint symptoms simultaneously with rashes or within 11 days of appearance of the rash. Forty-six cases (65 %) developed joint symptoms after having had the rash for 1 to 3 days. Nineteen cases (27 %) experienced the symptoms between 4 and 7 days, while four cases (6 %) experienced symptoms in the period between 8 and 11 days.

**Fig. 5:**
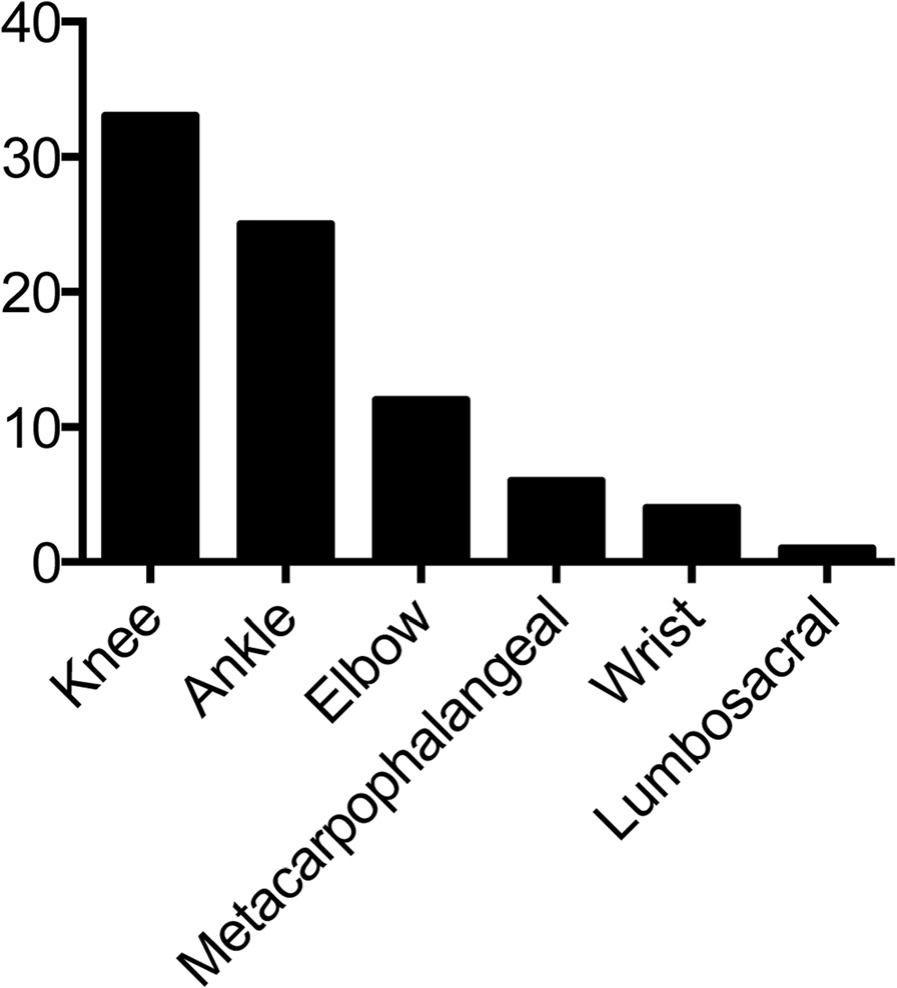
Distribution of affected joints. Knee and ankle joints were the most frequently affected

The joint manifestations included arthritis and arthralgia. Some children had sudden onset of symptoms during resting state. The arthralgia was worsened on extra weight bearing. A total of 71 (100 %) patients had arthralgia and discomfort, including 59 patients (83 %) with joint swelling, three patients (4 %) limited mobility in the absence of swelling; nine patients (13 %) had arthralgia only. Distending pain (pain accompanied by a distending sensation) was reported in 29 cases (41 %), sore pain (pain with sore and tired discomfort) in 22 cases (31 %), hot pain (hot and painful) [9] in 13 cases (18 %) and seven cases had indescribable nature of pain (10 %). Multiple joints were often involved (Fig. 5), and the pain was migratory and recurring in nature. In 46 cases (65 %), the joint involvement was unilateral, while 25 cases (35 %) had bilateral involvement. A total of 26 cases (37 %) experienced recurrence; 11 cases (15 %) recurred within 1 month, seven cases (10 %) recurred within 1 to 3 months, five cases recurred (7 %) within 3 to 6 months, three cases (4 %) relapsed in 6 to 17 months. Joint symptoms occurred simultaneously or successively with rash during relapse. Thirteen patients (18 %) relapsed on more than two occasions. However, the recurrence rate of joint involvement was relatively low as compared to that of rash. Joint symptoms usually improved within 1 to 5 days (median, 3 days); 29 cases improved within 1 to 2 days (41 %), 37 cases (52 %) in 3 to 4 days and five cases (7 %) improved in 5 days.

### Gastrointestinal symptoms

Forty patients (56 %) had gastrointestinal symptoms including abdominal pain, nausea, vomiting, with diarrhea or melena on few occasions. Children tended to have one or more of the above symptoms; 35 children (49 %) had abdominal pain, 21 (30 %) had vomiting, and 13 cases (18 %) had gastrointestinal bleeding. There were no cases with intussusception.

### Renal involvement

A total of 37 children (52 %) (23 males and 14 females with ratio at 1.64:1) had signs of renal involvement, mostly onset within one month of appearance of rash. In 19 cases (27 %), the renal involvement manifested within 15 days of appearance of the rash; in nine cases (13 %) onset from 16 days and 1 month, in five cases (7 %) between 1 and 3 months, in three cases (4 %) between 3 months and 1 year, and in one case (1 %) between 1 and 3 years. Renal involvement tended to manifest after skin symptoms. Proteinuria (11 %) was detected in eight cases, hematuria in six cases (8 %), concomitant hematuria and proteinuria in 13 cases (18 %), acute nephritis in 31 cases (44 %), nephrotic syndrome in 11 cases (15 %), and chronic nephritis in two cases (3 %).

Other symptoms were as follows. Leg edema was observed in 12 cases (17 %), fever in seven cases (10 %) (body temperature, 37.5 °C–38.7 °C). Five (7 %) patients suffered from testicular swelling and pain. Orchitis and testicular torsion was excluded by ultrasound examination. Three cases (4 %) had headache and dizziness. Cranial CT examination showed no abnormalities. In two cases, there were visible lung shadows which resolved after treatment.

### Laboratory tests

Results of antinuclear antibody, anti-DNA antibodies, and anti-E NA antibodies tests were negative. Total leukocytes count was increased in 39 cases (55 %) while the percentage of neutrophils were increased in 43 cases (61 %). Platelet counts and coagulation profile were normal. Occult blood was positive in 35 cases (49 %); CRP levels were found increased in 19 cases (27 %); anti-streptolysin O titers were increased in 14 cases (20 %) and C3 in 18 cases (25 %). IgA, IgG and IgM were increased in 13 (18 %), 3 (4 %) and one case (1 %), respectively. D-dimer was found increased in 34 cases (48 %). Knee effusion was observed on X-ray examination in three patients (4 %). Mesenteric lymph node enlargement was detected on abdominal ultrasonography in nine patients (13 %). In patients with upper respiratory tract infections, throat swab culture yielded beta-hemolytic streptococci and *Staphylococcus aureus* in 17 (24 %) and 12 (17 %) cases, respectively.

### Misdiagnosis and treatment

In two cases (3 %), joint symptoms appeared 6 and 13 days, respectively, prior to the onset of the rash and were treated in orthopedics department with immobilization and anti-infectives. In five (7 %) children abdominal pain was the first symptom and treated by anisodamine and cimetidine but with little symptom relief. A few days later, blood spots and petechiae with arthralgia appeared. One case (1 %) presented as acute abdomen pain suggestive of gastrointestinal obstruction. The rash appeared when the patient was being prepared for surgery. After consultation with our department, treatment of 80 mg (2 mg/kg per day) methylprednisolone was administered once a day for 3 days and the symptoms subsided.

General treatments included bed rest, avoidance of high protein diet and strenuous exercise. Temporary fasting and nutritional support therapies were advised for patients with severe gastrointestinal symptoms. Oral anti-histamine drugs, such as loratadine or cetirizine was administered. Patients with bacterial infections were given antibiotics, and leflunomide or tripterygium was administered in patients with renal involvement.

All 71 patients were treated with corticosteroids, such as methylprednisolone at 1–2 mg/kg per day as suggested by Joel et al. [10]. The treatment lasted from 1 to 4 weeks and the dosage was gradually reduced after 3 to 5 days, depending on the alleviation of symptoms. During the corticosteroids treatment, we monitored the patients for changes in rash, joint symptoms, and gastrointestinal manifestations. The average time for rash dissipation, reduction of joint swelling and pain, and gastrointestinal symptoms recovery (symptoms of abdominal pain, nausea under control, fecal occult blood test turned negative) were 5.76 ± 1.72, 2.65 ± 1.08, and 3.25 ± 1.28 days, respectively. Clinical and laboratory parameters, including BMI, blood pressure, routine blood test (WBC, RBC, PLT), liver function (ALT, AST) and kidney function (SCr, BUN) were measured before, during, and up to 4 weeks after corticosteroid treatment. Some patients had improvement in these values during corticosteroids treatment, though not significant compared to either pre-treatment or post-treatment (*P* > 0.05). Also, there was no significant difference in these values between pre-treatment and post-treatment periods (*P* > 0.05). In contrast to previous reports [11], patients who were treated with corticosteroids had faster recovery from rashes (t = −10.00, *P* = 0.00) and joint swelling and pain (t = −34.62, *P* = 0.00) than patients who did not have corticosteroids treatment.

### Clinical outcomes

The patients were followed up for 3 years. Fifty-two cases (73 %) had recurrence of rash. Kidney symptoms, joint symptoms, gastrointestinal symptoms were recurred in 29 cases (41 %), 26 cases (37 %) and 14 cases (20 %), respectively. Recurrence of abdominal pain, arthralgia and other symptoms tended to last for a short time. Some children experienced recurrence after withdrawal of treatment, episodes of upper respiratory tract infection or strenuous activity. Among the 37 cases of HSP nephritis, 19 cases (27 %) of proteinuria and urine occult blood turned negative within 1 week to 1 month of incidence, of which urine abnormality was detected in 13 cases (18 %) during first the 2 weeks; in another 15 cases (21 %) urine became normal within 1 month to 1 year and a half; in three cases (4 %) microscopic hematuria, and proteinuria continued to persist in small amounts.

On univariate analysis, gastrointestinal symptoms, increased levels of CRP and D-dimer and scrotal involvement were found to be significantly associated with renal impairment in HSP children with joint manifestations (Table 2). On multivariate logistic regression analysis, scrotal involvement, gastrointestinal symptoms, raised D-dimer levels were independently associated with renal impairment in these children (Table 3).

**Table 2:** Univariate analysis of risk factors for kidney injury [N (%)]

Risk factor	HSP (*N* = 34)	HSPN (*N* = 37)	χ^2^	*P* value
Male	17 (50 %)	23 (62 %)	1.07	0.30
Age ≥10 years	9 (26 %)	12 (32 %)	0.30	0.58
Rash ≥ 3 areas	11 (32 %)	16 (43 %)	0.89	0.35
Rash recurrence ≥ 3 times	8 (24 %)	14 (38 %)	1.70	0.19
Joints recurrence ≥ 2 times	5 (15 %)	8 (22 %)	0.57	0.45
Scrotum involvement	1 (3 %)	11 (30 %)	9.05	0.00
Gut involvement	13 (38 %)	27 (73 %)	8.69	0.00
IgA increase	7 (21 %)	12 (32 %)	1.27	0.26
D-dimer increase	9 (26 %)	25 (68 %)	11.99	0.00
CRP increase	5 (15 %)	14 (38 %)	4.84	0.03

*CRP* C-reaction protein*,* HSP Henoch-Schönlein purpura*, HSPN* HSP nephritis

**Table 3:** Independent predictors of renal damage on multivariate analysis

Risk factor	β	*P* value	OR	95 % CI
Scrotum involvement	3.65	0.00	38.42	3.48 - 423.77
Gut involvement	1.63	0.02	5.08	1.28 - 20.10
D-dimer increase	1.41	0.03	4.09	1.18 - 14.23
CRP increase	1.38	0.06	3.99	0.97 - 16.42

*CI* confidence interval, *CRP* C-reactive protein, *OR* Odds ratio

## Discussion

HSP has been reported to occur most frequently at age of 4 to 7 [12, 13] and it has also been reported to onset at around 6 to 10 years old [8, 14]. Although most studies have shown a male preponderance [8, 12–15], but there are reports where greater number of females has been found to be affected [16, 17]. In the present study, most of the cases of HSP with joint symptoms occurred between 6 to 11 years of age, more often in school-age children than in preschool children, and more in boys than in girls. This may be due to increased exposure to various allergens and a higher chance of contracting infection; especially in boys as they are generally more active than girls. Infection has been previously reported as the major predisposing factor for HSP [6, 12, 13], with most of the cases being reported in winter, fall and spring. Our data showed that respiratory tract infection was the major predisposing factor, with upper respiratory tract infection accounting for 32 % cases and lower respiratory tract infection accounting for 17 % cases. In this study, HSP was observed onset around all seasons with autumn and winter seasons being the most common. This may be due to dramatic seasonal temperature changes and children were prone to respiratory infections. Disease onset due to strenuous exercise was less common compared with previous report [18].

The diagnosis was usually straightforward in patients presenting with skin purpura as the initial symptom. In the present study, 90 % of the children developed skin manifestations at the outset. Most commonly, the rash first appeared in the lower limbs and foot; and gradually spread to other limbs. High relapse rate (73 %) was observed within 3 months and subsequently the recurrence rate gradually declined, which might be due to the gradual weakening of the aberrant immune response. In previous studies, rash has been reported to be occurring mainly in lower limbs and buttocks [9]. Most children have been reported to develop a recurrent rash within 12 months, with lesions relapsing one to five times, and late recurrence being associated with milder symptoms that usually lasted for a short duration [13].

Seventy-one children had varying degrees of joint involvements, which occurred with rash in most patients or after rash with no specific clinical manifestations. Patients with joint symptoms as initial manifestations were easily confused with rheumatoid arthritis, sprains and etc. However, anti-infection treatment was effective, which also confirmed HSP with joint symptoms were associated with infection. Pain, swelling and other symptoms sometimes did not occur simultaneously, mostly occurred as acute arthritis with different nature of pain. Similar to other studies, we also found that the joint symptoms frequently occurred in weight bearing large joints with knee and ankle joints being the most likely to be affected, since these joints move more and they are more vulnerable to injuries. In our study, joint symptoms could relapse with rash, mostly unilateral, but Trapani et al. reported that the rash could also be symmetrical [13].

Abdominal pain, vomiting and gastrointestinal bleeding are the most common symptoms in patients with HSP [6, 8, 13, 14, 17]. The reported incidence of gastrointestinal symptoms could be as high as 34 to 75 %. However, some reports have indicated gastrointestinal bleeding as the most common symptom [13, 17] while in some other reports pain was the most common symptom [13, 14]. In our study, 56 % of all patients had gastrointestinal symptoms, with abdominal pain being the most common, followed by vomiting and gastrointestinal bleeding. The pain was often periumbilical or in the upper abdomen as presented as paroxysmal colic.

In our study, 11 % of children had abdominal pain or arthralgia as the initial clinical presentation, which resulted in misdiagnosis. Although it was lower than previously reported [7, 8, 14], in 15 to 25 % of patients, joint symptoms appeared one week prior to the rash. Ten to 20 % of these patients have been reported to present with abdominal pain two weeks prior to the appearance of rash. Our experience suggests that if children with abdominal pain or arthralgia are admitted and develop rash a few days later, the diagnosis of HSP should be considered. Thus, it is important for the health care professionals to have adequate knowledge about HSP so as to avoid unnecessary invasive procedures in such cases.

Renal function is a key determinant of prognosis in HSP patients. Twenty to 54 % of HSP children may develop renal involvement [13, 19–21]. Similar to that reported by Trapani et al., 52 % children in our study had renal involvement [13] Although the exact mechanism of pathogenesis is not clear, previous studies have implicated IgA-containing immune complex deposition in glomerular basement membrane [4]. The main pathological changes include mesangial proliferative changes, with varying degrees of segmental necrosis and glomerular crescent formation [22] Earlier studies have shown that HSP nephritis (HSPN) is mostly seen within 1 month, with some cases occurring within 6 months, and a few cases appearing after 1 year. Among these cases, 1 to 2 % of patients may develop irreversible kidney damage [23] In the present study, 76 % cases developed HSPN within 1 month, with most occurring within 2 weeks. There were no cases of end-stage renal disease observed during the 3-year follow up period. The prognosis for HSPN is good [5, 12, 13].

Our multivariate analysis, scrotal involvement, gastrointestinal symptoms and raised D-dimer levels significantly correlated with kidney damage. Compared to patients without these above risk factors, presence of these factors was associated with 38.42 times, 5.08 times, 4.09 times higher incidence rates, respectively. Tabel et al. [24] also reported scrotal involvement as a risk factor for renal impairment. A study Elmas et al. [25] also showed an association of gastrointestinal symptoms with kidney damage. Yilmaz et al. [26] reported that D-dimer levels in HSP patients with renal damage were significantly higher than in those without renal involvement. Similar findings were also reported by another study [27].

In the present study, HSP patients with joint symptoms readily responded to methylprednisolone treatment. No long-term side effects were observed and treatment compliance of patients tended to be good. Not only the effect of glucocorticoids on joints and abdominal symptoms in HSP has been confirmed [28, 29], but other studies have also reported its role in preventing kidney disease [30, 31]. With timely glucocorticoids in optimal dosage, adverse reactions tend to be reversible. Titrating the dose to the severity of the symptoms appears to be the key paradigm in successful management of children with HSP. One of the limitations of this study is the relatively small sample size due to availability of patients. The sample collection process lasted for 3 years. Due to limitation by experience and time and space, the present study does not allow conclusions about therapeutic approaches, which should be made from prospective controlled studies.

## Conclusion

To summarize, HSP with joint involvement most commonly occurred in school-aged males during fall and winter seasons. Upper respiratory tract infection was the predominant triggering factor. Gastrointestinal symptoms, raised D-dimer levels and scrotal involvement were found to be independently associated with renal involvement. Rational use of corticosteroids significantly alleviated the symptoms, with no long-term adverse effects being observed during follow-up. The present study does not answer questions including why some patients experienced joint involvement and others suffered from gastrointestinal damage and how to explain the increase of D-dimer occurred only in some patients and others did not.

## Abbreviation

HSP: Henoch-Schönlein purpura
